# DISCRIMINATION AND COGNITIVE HEALTH DISPARITIES AMONG OLDER AMERICANS: A FOCUS ON NATIVITY AND RACE/ETHNICITY

**DOI:** 10.1093/geroni/igae098.3448

**Published:** 2024-12-31

**Authors:** Yi Wang, Man Guo, Huei-Wern Shen, Morgan Stangl

**Affiliations:** University of Iowa, Iowa City, Iowa, United States; University of Iowa, Iowa City, Iowa, United States; University of North Texas, Denton, Texas, United States; University of Iowa, Iowa City, Iowa, United States

## Abstract

Discrimination, characterized by unfair or prejudicial treatment based on perceived differences, has been identified as a significant stressor with potential detrimental effects on cognitive health in the aging population. Disproportionately experienced by immigrants and racial/ethnic minorities, discrimination can exacerbate existing health disparities. Using data from the 2016 and 2018 Leave-Behind Questionnaire of the Health and Retirement Study (N=9,440), this study aims to examine 1) What is the associations between discrimination and cognitive health (global cognitive functioning score), and whether such relationships vary by nativity (US born: yes/no)? 2) Does race/ethnicity (White, Black, Hispanic) moderate the above relationships? and 3) Do contextual resources (neighborhood social cohesion, social support from family and friends) and activity engagement (volunteering, cognitively stimulating activities) serve as protective factors for cognitive health in the face of discrimination? Results from linear regression models revealed that discrimination is negatively linked to cognitive health and this relationship is stronger for immigrants than non-immigrants. Across nativity and race/ethnicity groups, only Hispanic immigrants reported a significantly lower cognitive score (in comparison with U.S. born Whites) when facing higher levels of perceived discrimination. As for protective factors, both activity engagement indicators were associated with higher cognitive scores among immigrants. These findings emphasize the heightened vulnerability of immigrant populations, particularly Hispanic immigrants, to the negative effects of discrimination on cognition. The protective factors, such as volunteering and engaging in cognitively stimulating activities, highlights avenues for intervention and support to mitigate the adverse consequences of discrimination on cognitive health among older immigrants in the U.S.

